# Supplementary material for: Dysregulation of Placental Lipid Hydrolysis by High-Fat/High-Cholesterol Feeding and Gestational Diabetes Mellitus in Mice
Source: Int J Mol Sci. 2022 Oct 14;23(20):12286. doi: 10.3390/ijms232012286 (PMC9603336; doi:10.3390/ijms232012286)
Supplement: Supplementary file 1 [file ijms-23-12286-s001.zip › ijms-1927724-supplementary.pdf]

SUPPLEMENT

Dysregulation of placental lipid hydrolysis by high-fat/high-cholesterol feeding and gestational diabetes mellitus in mice" by K.B. Kuentzel *et al.*

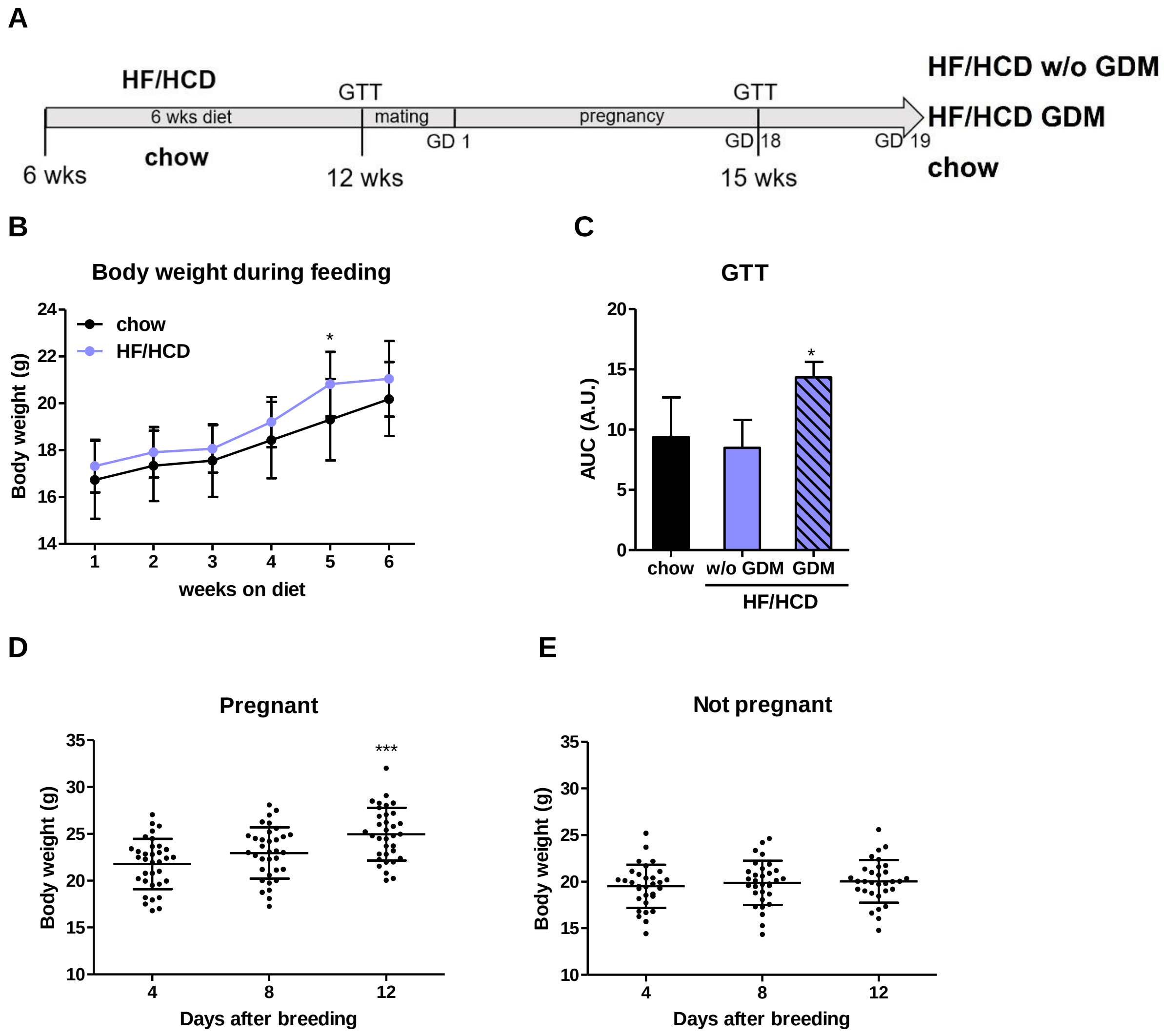

Figure S1: Generation of the GDM mouse model. (A) Experimental setup and timeline for the generation of a GDM mouse model; HF/HCD – high fat/high cholesterol diet; GTT – glucose tolerance test; GD – gestational day. (B) Body weight during HF/HCD feeding prior to mating. (C) Area under the curve (AUC) of the GTT of pregnant mice (n = 3 chow, 8 HF/HCD w/o GDM, 4 HF/HCD GDM). (D, E) Pregnancy detection in mice according to maternal weight gain, n = 34 (not pregnant), 31 (pregnant). Data represent mean ± SD and were analyzed with 1-way ANOVA followed by Bonferroni post-hoc test. * $p < 0.05$, *** $p \leq 0.001$.

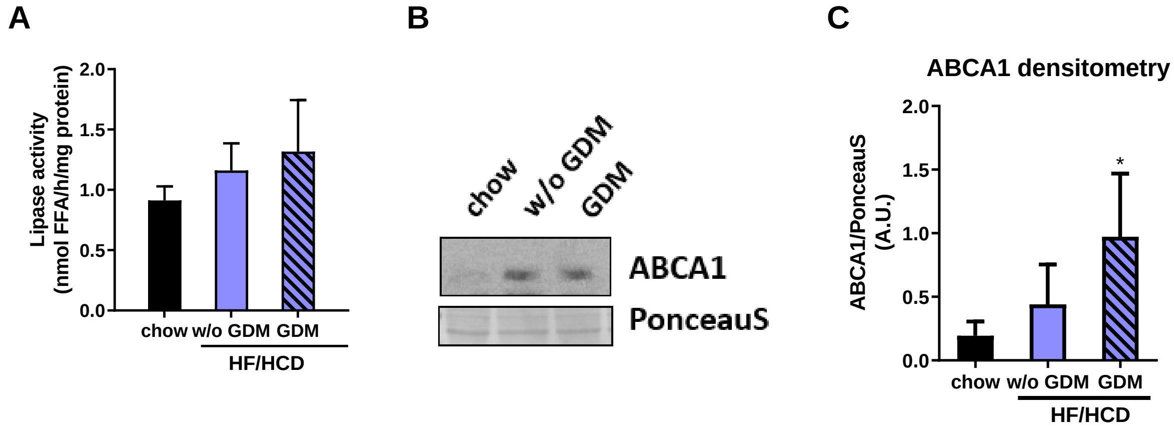

Figure S2: Increased placental cholesterol export in GDM. (A) Acid CE hydrolase activity in placentae of chow, HF/HCD w/o GDM, and HF/HCD GDM dams (n = 4-5). (B) Western blot analysis of ABCA1 and (C) densitometry in placentae of chow, HF/HCD w/o GDM, and HF/HCD GDM dams, representative blot shown (n = 2). Data represent mean + SD and were analyzed with 1-way ANOVA followed by Bonferroni post-hoc test. * $p < 0.05$.

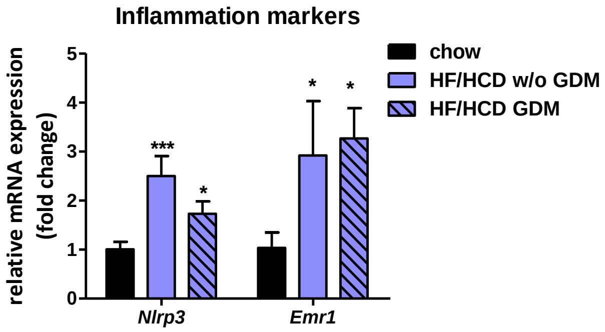

Figure S3: Increased inflammation and macrophage abundance accompanying HF/HCD feeding and GDM. Gene expression levels of inflammatory (*Nlrp3*) and macrophage (*Emr1*) markers in placentae from chow diet-fed mice and after HF/HCD feeding w/o and with GDM (n = 4). Data represent mean + SD and were analyzed with 1-way ANOVA followed by Bonferroni post-hoc test. * $p < 0.05$, *** $p \leq 0.001$, indicating the comparison to the chow condition.

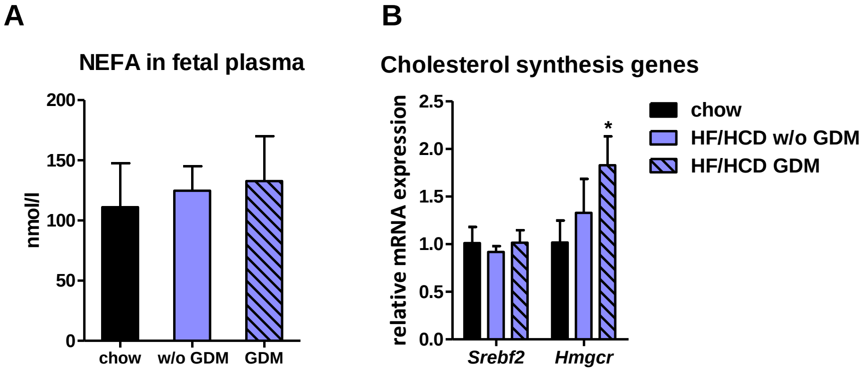

Figure S4: Unaltered fetal plasma FA levels upon HF/HCD feeding and GDM. (A) Concentrations of non-esterified FA (NEFA) in the plasma of chow diet, HF/HCD without and with GDM mice (n = 4). (B) mRNA expression of cholesterol synthesis genes (n = 4). Data represent mean + SD and were analyzed with 1-way ANOVA followed by Bonferroni post-hoc test. * $p < 0.05$
